# CEMIP induces TGF-β/Smad signaling to promote keloid development by binding to SPARC

**DOI:** 10.1016/j.clinsp.2024.100523

**Published:** 2024-10-30

**Authors:** Xinyi Li, Wei Zhang, Xiaojing Li

**Affiliations:** Department of Plastic Surgery, First Affiliated Hospital of Anhui Medical University, Hefei, China

**Keywords:** CEMIP, SPARC, TGF-β/Smad, Keloid Hyperplasia, Keloid Fibroblasts

## Abstract

**Background:**

Cell Migration Inducing Hyaluronidase 1 (CEMIP) is a protein that plays regulatory functions in a variety of cellular processes in many diseases. Nevertheless, its role and molecular mechanism in keloid hyperplasia are still elusive.

**Methods:**

Expressions of CEMIP and Secreted Protein acidic and Rich in Cysteine (SPARC) were detected by qRT-PCR and western blot. CCK-8 assay, along with immunofluorescence staining, was applied for the assessment of cell proliferation. The capabilities of cells to migrate and invade were evaluated utilizing wound healing and Transwell, while Extracellular Matrix (ECM) deposition was measured by immunofluorescence and western blot. The interaction of CEMIP and SPARC was predicted by the Coexpedia and PPA-red databases and verified by co-IP. Western blot was adopted for the estimation of TGF-β/Smad pathway-related proteins.

**Results:**

The data demonstrated that CEMIP expression was elevated in Keloid Fibroblasts (KF). CEMIP interference suppressed cell proliferative, migrative and invasive capabilities and ECM deposition in KF. Mechanistically, bioinformatics analysis revealed that CEMIP was co-expressed with SPARC and CEMIP protein could bind to SPARC. SPARC expression was reduced in CEMIP-silenced cells. SPARC overexpression counteracted the impacts of CEMIP silencing on cell proliferative, migrative and invasive capabilities and ECM deposition in KF. In addition, the expressions of TGF-β/Smad signaling-related proteins were decreased by CEMIP silencing via the inhibition of SPARC.

**Conclusion:**

In summary, this study revealed that CEMIP modulated KF proliferation, migration, invasion and ECM deposition by TGF-β/Smad signaling through binding to SPARC.

## Highlights

• CEMIP silencing suppresses the proliferation, migration, invasion and ECM deposition of KF.
• CEMIP binds to SPARC.
• SPARC overexpression reversed the effects of CEMIP silencing on KF.
• The TGF-β/Smad signaling was associated with the regulation of CEMIP SPARC in KF.

## Introduction

As a benign fibrous tumor of the skin, keloid results from the over-proliferation of fibroblasts as well as excessive deposition of Extracellular Matrix (ECM) in the dermis after skin injury.^1^^,^^2^ The pathogenesis of keloid is complex, which may be related to genetic, immune as well as cellular signal transduction pathways.^3^ Keloid scar not only influences beauty, but also contributes to picking pain, pruritus and even cause dysfunction.^4^ The treatment approaches to keloid predominantly include surgery, local medication, cryotherapy, and a combination of laser and radiation therapy.^5^ Although keloid is a benign dermal tumor, the treatment outcome of keloid patients is not ideal because of its high recurrence. With an in-depth understanding of the genetic and immune correlation of keloid, other therapeutic methods have been developed, such as gene therapy, targeted therapy, and immunotherapy, but the therapeutic effects of these treatment methods have yet to be verified.^6^^,^^7^

Being an intranuclear protein, Cell Migration-inducing hyaluronidase 1 (CEMIP) is located in human chromosome 15q25.1 with the enzymatic capacity to degrade hyaluronan.^8^ An increasing number of studies have demonstrated that CEMIP has abnormal expression in breast cancer, colon cancer, prostate cancer, and other tumors, which can promote the biological process of tumor invasion, metastasis, apoptosis and so on.^9^, ^10^, ^11^ CEMIP could promote Epithelial-Mesenchymal Transition (EMT) process in colorectal cancer and facilitate mesenchymal transformation shuttling into circulation.^12^ Also, another study reported that CEMIP promoted cell invasion and migration and triggered EMT of Non-Small-Cell Lung Cancer (NSCLC) cells through PI3K/AKT pathway.^13^ In addition, CEMIP was reported to participate in the enhanced degradation of Hyaluronic Acid (HA) in dermal fibroblast and Wnt/β-catenin signaling pathway.^14^ Moreover, it was also evidenced that CEMIP was upregulated in synovial fibroblasts in Rheumatoid Arthritis (RA) or Osteoarthritis (OA) patients.^15^ All the above-mentioned findings indicate that CEMIP acts as a critical regulator in cell proliferation, invasion, migration and fibrosis, but its role in keloid hyperplasia has not been studied. Thus, this study was designed, aiming to identify the biological roles of CEMIP in keloid hyperplasia and to disclose its potential mechanisms.

## Materials and methods

### Bioinformatic analysis

Coexpedia database (https://www.coexpedia.org/search.php) was used to predict the co-expression of CEMPI and Secreted Protein Acidic and Rich in Cysteine (SPARC). PPA-red (https://www.iitm.ac.in/bioinfo/PPA_Pred/prediction.html#) predicted the combination of CEMPI and SPARC.

### Cell culture and treatment

Keloid Fibroblasts (KF) and normal fibroblasts (NF) that provided by the American Type Culture Collection (Rockville) were cultivated in DMEM (Invitrogen) supplemented with 10% FBS and 1% penicillin-streptomycin with 5% CO_2_ at 37 °C.

### Cell transfection

For the knockdown of CEMIP, specific shRNA targeting CEMIP (shRNA-CEMIP-1/2), and corresponding control shRNA (shRNA-NC) were synthesized by Gene Pharma (Shanghai, China). To overexpress SPARC, the pc-DNA 3.1 vector containing the whole length of SPARC (Ov-SPARC) and the empty vector (Ov-NC) were synthesized by Shanghai Genechem Co., Ltd. 100 nM recombinants were transfected into KF utilizing Lipofectamine 2000 (Invitrogen-Life Technologies) in light of standard protocol. After transfection for 48h, KF were collected for ensuing research.

### Cell counting kit-8 (CCK-8) assay

KF with indicated treatment were inoculated into 96-well plates and then cultivated in DMEM with 10% FBS for 24h, 48h and 72h Afterwards each well was added with 10 μL WST-8 (Beyotime) to further incubate the plates for 2h, and then the absorbance was appraised by means of a microplate reader (Bio-Rad) at 450 nm.

### Immunofluorescence staining

The transfected KF were subjected to 4% polyoxymethylene fixation and 0.5% Trition-X100 permeation. Following the block with 10% BSA in PBS, the overnight subjection of KF to primary antibodies against Ki67 and FN was conducted at 4 °C, after which was the cultivation with secondary antibodies for 1h at room temperature. DAPI was applied for the counterstaining of KF and the observation was implemented under a fluorescence microscope (Nikon Eclipse80i).

## Wound healing assay

KF with indicated treatment were inoculated into 6-well plates and then cultured until 80%–90% cell fusion was reached. With the help of white pipette tips, wound-in cell monolayers were made. Then, the PBS-rinsed cells were cultured in a serum-free medium for 24h at 37 °C. Finally, the wounds were recorded at 0 and 24h utilizing an inverted microscope (Olympus Corp).

### Transwell assay

KF with indicated treatment were inoculated into the serum-free medium (200 μL) in the upper chambers pre-coated with Matrigel. RPMI-1640 medium that was decorated with 10% FBS was loaded in the lower chamber. After incubation for 24h, invaded cells on the lower face were subjected to 100% methanol fixation and H&E staining. The images of cells passing through the membranes were captured utilizing an inverted microscope. Five random fields were selected for counting.

### Co-immunoprecipitation (co-IP)

Lysates that were prepared from cells using lysis buffer (150 mM NaCl, 10 mM HEPES, pH 7.4, 1% NP-40) were then exposed to HA affinity agarose (Sigma-Aldrich), or CEMIP or SPARC antibody with protein G agarose overnight at 4 °C. Beads containing affinity-bound proteins were washed 6 times by immunoprecipitation wash buffer (150 mM NaCl, 10 mM HEPES, pH 7.4, 0.1% NP-40), following which was the elution with 1 M glycine (pH 3.0). The eluates were then mixed with sample buffer, denatured, and applied for analysis by western blot.

### RNA extraction and quantitative real-time PCR (qRT-PCR)

Total RNAs that isolated from sample KF with Trizol reagent (Invitrogen) were reverse transcribed into cDNA utilizing PrimeScript RT Master Mix (Perfect Real Time; Takara Bio, Inc.) in light of standard protocol. The templets were amplified on an ABI PRISM 7900 Real-Time system (Applied Biosystems) by virtue of the SYBR Premix ExTaq kit (Takara Bio, Inc.). The primer sequences for PCR are presented as below: CEMIP: 5′-ACCCATCACTCGGTCTCTGA-3′ (forward) and 5′-GAGGTGAGCAGCAGTGTCTT-3′ (reverse); SPARC: 5′-CAAGAAGCCCTGCCTGATGA-3′ (forward) and 5′-TCTTCGGTTTCCTCTGCACC-3′ (reverse); GAPDH: 5′-GGGAAACTGTGGCGTGAT-3′ (forward) and 5′-GAGTGGGTGTCGCTGTTGA-3′ (reverse). Data were demonstrated in the format of fold change (2^-ΔΔCt^), which normalized to GAPDH.

### Western blot

Total proteins that were isolated from sample KF with RIPA buffer (Auragene). Following the separation with 10% SDS-PAGE, the transfer of proteins to PVDF membranes was implemented. Membranes, which were impeded by 5% BSA, were incubated with antibodies targeting CEMIP, Collagen I, α-SMA, FN, SPARC, TGF-β, p-SMAD2, p-SMAD3, SMAD2, SMAD3 and GAPDH at 4 °C overnight. After that, the PBST-rinsed membranes were probed with appropriate secondary antibodies for 1h at room temperature. Finally, the visualization of protein bands was implemented employing an ECL detection system (Beyotime) and ImageJ software (Version 1.49) was adopted to analyze protein density.

### Statistical analysis

Data collected from three independent experiments were displayed in the format of mean ± SD and were analyzed utilizing SPSS 19.0 software (Chicago). For differences between the two groups, Student's *t*-test was adopted while one-way ANOVA with a post hoc Bonferroni multiple comparison test was employed for the comparisons among multiple groups. P less than 0.05 was viewed to have statistical significance.

## Results

### Downregulation of CEMIP suppresses KF proliferation, migration and invasion

**Fig. 1:**
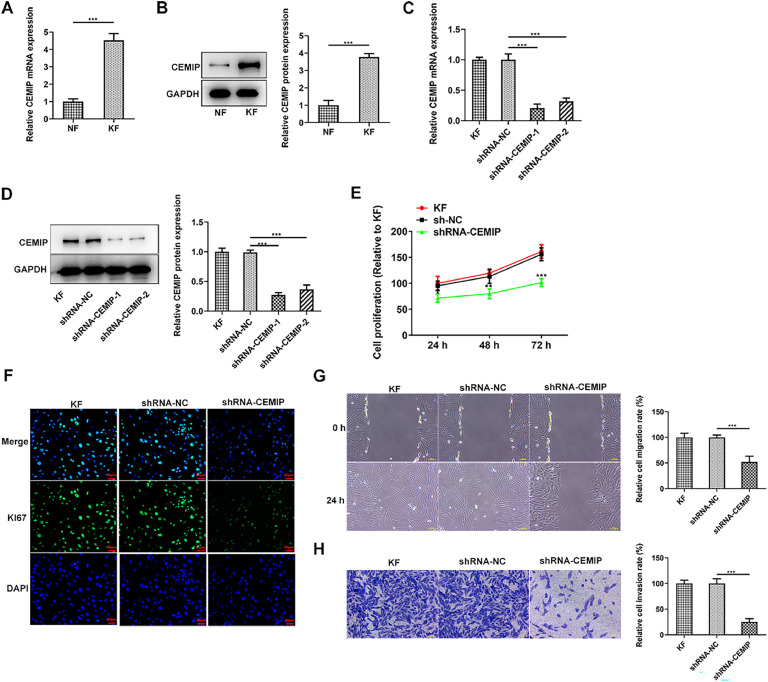
Downregulation of CEMIP suppresses KF proliferation, migration and invasion. The mRNA (A) and protein (B) levels of CEMIP in Keloid Fibroblasts (KF) and Normal Fibroblast (NF) were detected by qRT-PCR and western blot. The mRNA (C) and protein (D) levels of CEMIP in KF transfected with shRNA-CEMIP were detected by qRT-PCR and western blot. (E) Cell proliferation was evaluated by CCK-8 assay. (F) Immunofluorescence staining was used to detect the level of Ki67. (G) Cell migration was evaluated by wound healing assay. (H) Cell invasion was evaluated by transwell assay. Data are expressed as mean ± SD (*p < 0.05, **p < 0.01, ***p < 0.001).

With the purpose of investigating the role of CEMIP in keloid hyperplasia, CEMIP expression in keloid fibroblasts was initially assessed. As Fig. 1A‒B demonstrated, the mRNA and protein expressions of CEMIP in KF were conspicuously elevated relative to the Normal Fibroblasts (NF). To identify the biological role that CEMIP played in KF, CEMIP was silenced and qPCR as well as western blot was adopted for the examination of transfection efficiency (Fig. 1C‒D). Results obtained from CCK-8 assay depicted that CEMIP silencing remarkably inhibited cell proliferation (Fig. 1E). In addition, immunofluorescence staining showed that the level of Ki67 in KF was reduced by CEMIP silencing (Fig. 1F). Moreover, the knockdown of CEMIP reduced the rate of cell migration when compared with the shRNA-NC group (Fig. 1G). Transwell assay results indicated that the invasive ability in KF was restrained by the knockdown of CEMIP (Fig. 1H).

### CEMIP silencing inhibits ECM deposition in KF

**Fig. 2:**
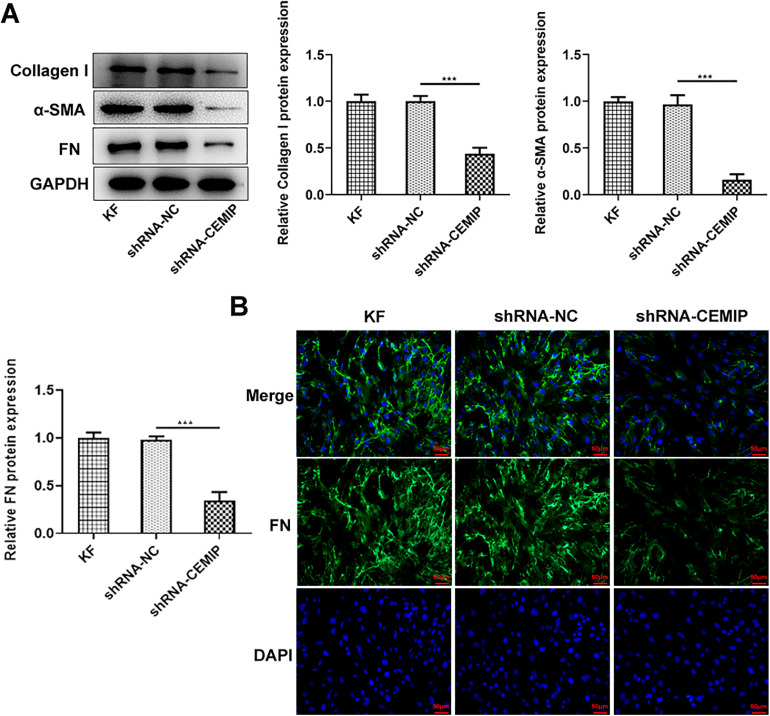
CEMIP silencing inhibits ECM deposition in KF. (A) The protein levels of Collagen I, α-SMA and FN in KF transfected with shRNA-CEMIP were assessed by western blot. (B) Immunofluorescence staining was used to detect the level of FN in KF transfected with shRNA-CEMIP. Data are expressed as mean ± SD (*** p < 0.001).

Then, the authors explored the effects of CEMIP silencing on ECM deposition in KF. As shown in Fig. 2A, CEMIP knockdown specifically decreased the protein levels of Collagen I, α-SMA and FN by contrast with the shRNA-NC group. Consistently, data from immunofluorescence staining revealed that the level of FN in cells transfected with shRNA-CEMIP was markedly reduced (Fig. 2B).

### CEMIP binds to SPARC

**Fig. 3:**
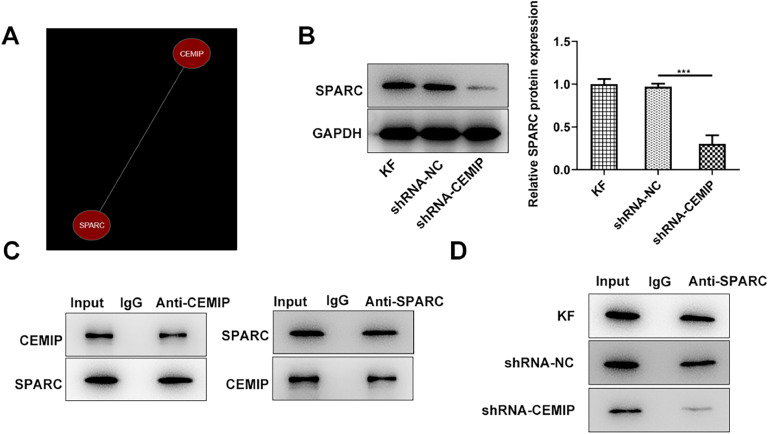
CEMIP binds to SPARC. (A) Coexpedia database revealed that CEMIP was co-expressed with SPARC. (B) The protein level of SPARC in KF transfected with shRNA-CEMIP was detected by western blot. (C) co-IP assay confirmed the binding of CEMIP and SPARC. (D) IP assay predicted that CEMPIP silencing inhibited SPARC precipitation. Data are expressed as mean ± SD (***p < 0.001).

The authors further explored the mechanism involved in the function of CEMIP in KF. As shown in Fig. 3A, Coexpedia database revealed that CEMIP was co-expressed with SPARC. PPA-red database also showed that CEMIP protein could bind to SPARC and predicted that the value of binding free energy is -15.51 kcal/moL. CEMIP silencing led to downregulated SPARC expression in KF (Fig. 3B). Moreover, co-IP assay confirmed the binding of CEMIP and SPARC (Fig. 3C). IP experiment also predicted that CEPIP silencing inhibited SPARC precipitation (Fig. 3D).

### CEMIP silencing restrains the proliferation, invasion and migration of KF by inhibiting SPARC

**Fig. 4:**
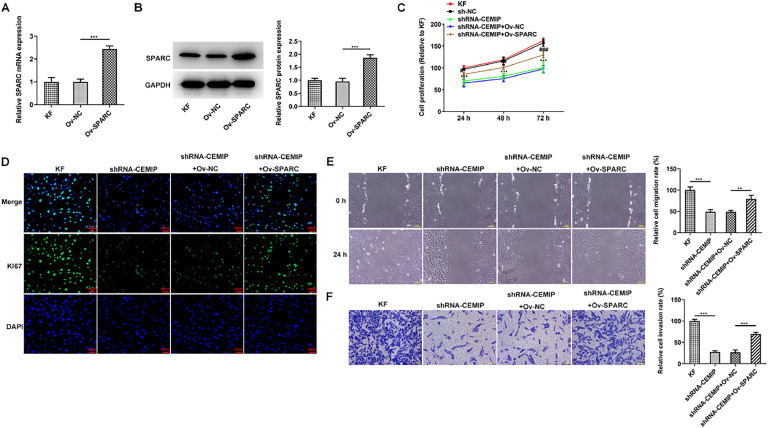
CEMIP silencing restrains the proliferation, invasion and migration of KF by inhibiting SPARC. The mRNA (A) and protein (B) levels of SPARC in KF transfected with Ov-SPARC were detected by qRT-PCR and western blot. (C) Cell proliferation was evaluated by CCK-8 assay. (D) Immunofluorescence staining was used to detect the level of Ki67. (E) Cell migration was evaluated by wound healing assay. (F) Cell invasion was evaluated by transwell assay. Data are expressed as mean ± SD (** p < 0.01, *** p < 0.001, # p < 0.05, ## p < 0.01, ### p < 0.001).

To identify the role of SPARC in CEMIP-mediated KF, Ov-SPARC was transfected into KF to elevate SPARC expression. The transfection efficiency of Ov-SPARC was examined utilizing qPCR as well as western blot (Fig. 4A‒B). As Fig. 4C depicted, SPARC overexpression elevated the reduced cell proliferation in CEMIP-silenced KF. Similarly, immunofluorescence assay revealed that Ki67 level in shRNA-CEMIP+Ov-NC group was significantly increased after the transfection with Ov-SPARC (Fig. 4D). Moreover, wound healing and transwell assay displayed that the decreased migration and invasion in shRNA-CEMIP+Ov-NC group were rehabilitated after SPARC was overexpressed (Fig. 4E‒F).

### Knockdown of CEMIP ameliorates ECM deposition in KF via binding to SPARC

**Fig. 5:**
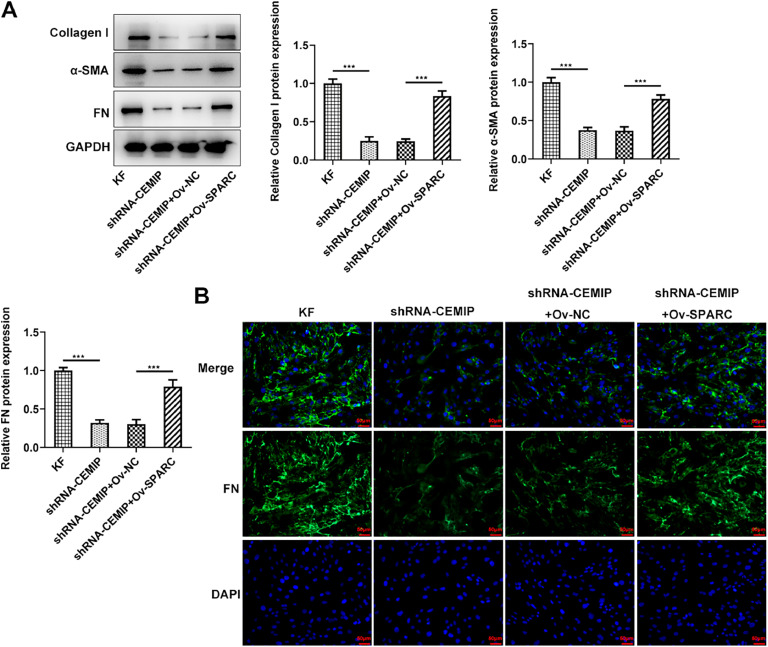
Knockdown of CEMIP ameliorates ECM deposition in KF via binding to SPARC. (A) The protein levels of Collagen I, α-SMA and FN in KF transfected with shRNA-CEMIP with the presence or absence of Ov-SPARC were assessed by western blot. (B) Immunofluorescence staining was used to detect the level of FN in KF transfected with shRNA-CEMIP with the presence or absence of Ov-SPARC. Data are expressed as mean ± SD (*** p < 0.001).

Next, the authors identify the role of SPARC in ECM deposition of CEMIP-mediated KF. As shown in Fig. 5A, CEMIP silencing abated the production of Collagen I, α-SMA and FN in KF compared with those in KF without transfection. However, SPARC overexpression counteracted the impacts of CEMIP silencing on these proteins. Consistently, the immunofluorescence assay revealed that the level of FN in KF was significantly decreased by CEMIP silencing, which was subsequently increased after the transfection with Ov-SPARC (Fig. 5B).

### CEMIP silencing blocks TGF-β/Smad signaling by suppressing SPARC

**Fig. 6:**
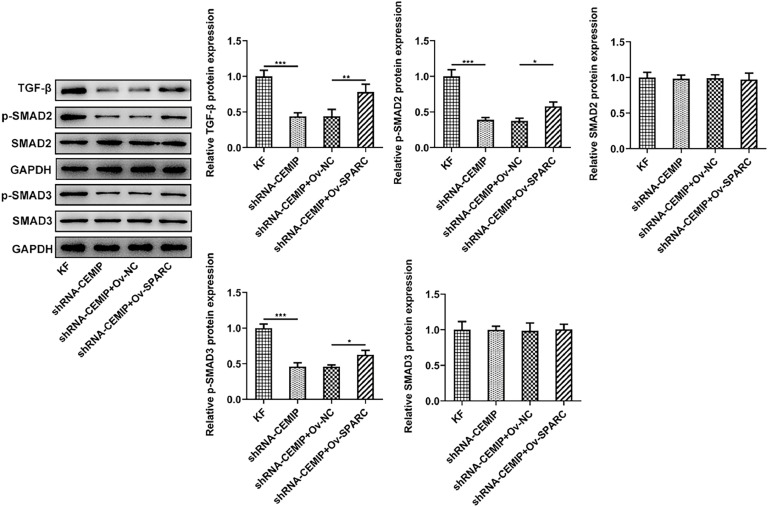
CEMIP silencing blocks TGF-β/Smad signaling by suppressing SPARC. Western blot was used to measure the levels of TGF-β, Smad2, p-Smad2, Smad3and p-Smad3 in KF transfected with shRNA-CEMIP with the presence or absence of Ov-SPARC. Data are expressed as mean ± SD (*** p < 0.001).

With the aim of further investigating the function of a downstream pathway of CEMIP in KF, a western blot was implemented. The results showed that CEMIP silencing significantly inhibited the level of TGF-β and the phosphorylation levels of Smad2 and Smad3 in KF, which were then reversed after overexpressing SPARC expression (Fig. 6).

## Discussion

Keloid is the result of excessive local tissue fibrosis after skin injury and wound healing.^16^ It is characterized by aggressive growth of fibroblasts and excessive deposition of ECM.^17^ Keloid is prone to recurrence and epitaxial growth without comprehensive anti-scar therapy after surgical resection.^18^ KF are flat and long spindle-shaped, with an obvious proliferation of spinous cells and increased cell layers in vivo, and are often considered to be the key cells of keloid formation.^19^ Fibroblasts are the main cell population in the lesional area, which proliferate and secrete a large amount of ECM, resulting in excessive collagen synthesis and deposition.^20^ Therefore, finding therapies or drugs to inhibit fibroblast proliferation and ECM may be an effective way to treat keloids. In this study, the authors demonstrated the promotive role of CEMIP and its relationship with SPARC in KF.

Previous studies have claimed that CEMIP acts as a stimulator in tumor cell growth, invasion, and spread of the tumors.^21^ For example, CEMIP enhanced the proliferative and migrative capabilities of breast cancer and prostate cancer cells.^22^^,^^23^ Moreover, CEMIP was reported to be increased in human and mouse inflamed synovial membranes and induce EMT pathway and fibrotic markers.^24^ Schmaus et al. revealed that Sulfated HA regulated the hyaluronan metabolism, proliferation and differentiation of fibroblasts by suppressing the hyaluronidase CEMIP.^25^ Deroyer et al. also reported that CEMIP was associated with the dedifferentiation of human chondrocyte into fibroblast-like chondrocytes and that CEMIP promoted α-SMA expression and TGF-β signaling towards the p-Smad2–3/Alk5/PAI-1 pathway.^26^ In the present study, the authors found that CEMIP was highly expressed in KF. CEMIP depletion repressed the capabilities of KF to proliferate, invade and migrate as well as suppressed the deposition of ECM.

The matricellular protein SPARC has been proven to participate in scar formation and tissue fibrosis.^27^ Previous studies have implicated that the depletion of SPARC in mice or rats markedly alleviated dermal, renal, pulmonary, or hepatic fibrotic processes.^28^^,^^29^ Lin et al. found that SPARC expression was increased in keloid dermal tissue.^30^ In addition, SPARC led to an increase in collagen matrix contraction and cell proliferation, thus promoting excessive wound healing and scar formation in human Tenon's capsules after filtration surgery.^31^ Through the present research, it was discovered that CEMIP can be co-expressed with SPARC and CEMIP can bind to SPARC. With the aim of further confirming the relationship between these two proteins, co-IP was performed to verify the combination of CEMIP and SPARC. It was implicated that SPARC overexpression counteracted the suppressive impacts of CEMIP interference on KF cell proliferation, invasion, migration, as well as ECM deposition.

Transforming Growth Factor-β (TGF-β) is viewed to be a critical fibrotic cytokine.^32^ TGF-β1 can directly or indirectly regulate the proliferative or apoptotic capability of fibroblasts, and then affect the formation of fibroblasts.^33^ TGF-β1 can also regulate collagen synthesis and degradation by activating or inhibiting fibroblastic type I and type III collagen promoters.^34^ Meanwhile, by down-regulating the secretion of collagenase and promoting the expression of its protein inhibitor, TGF-β1 suppressed the degradation of collagen and other ECM proteins, and stimulated the synthesis of ECM-receptor integrin to make cells adhere to matrix proteins.^35^ TGF-β1 phosphorylates intracellular Smad2 and Smad3 by binding to TGF-βI and II receptors on the cell membrane. Activated Smad2/Smad3 complex binds to Smad4 to enter the nucleus, and then affects fibroblast proliferative or apoptotic capabilities, collagen synthesis or degradation through gene regulation.^36^ A current Study has shown that SPARC induced TGF-β signaling promotes pro-fibrotic activation of systemic sclerosis patient dermal fibroblasts.^37^ In this study, the authors also explored the effects of CEMIP/SPARC on TGF-β1/Smad in KF. The data showed that CEMIP silencing specifically restrained the expression of TGF-β1 and the levels of p-Smad2 and p-Smad3, which were subsequently reversed by SPARC overexpression.

## Conclusion

In summary, this study disclosed that CEMIP silencing repressed KF proliferative, migrative and invasive capabilities, as well as suppressed ECM deposition, which may rely on the modulation of CEMIP-mediated TGF-β1/Smad pathway. These results may provide novel insights into keloid pathogenesis and develop therapeutic strategies for patients with keloid.

## Availability of data and materials

All data generated or analyzed during this study are included in this published article.

## Ethics approval and consent to participate

Not applicable.

## Patient consent for publication

Not applicable.

## Authors’ contributions

Xinyi L and Xiaojing Li designed the study, drafted and revised the manuscript. Xinyi Li and Wei Zhang analyzed the data and searched the literature. All authors performed the experiments. All authors read and approved the final manuscript.

## Funding

This work was supported by the 10.13039/501100003995Natural Science Foundation of Anhui Province, China (1908085QH327) and the Scientific research project of colleges and universities in Anhui province (2022AH051191).

## Declaration of competing interest

The authors declare no conflicts of interest.
